# Factors Influencing Nursing Students’ Person-Centered Care

**DOI:** 10.3390/medicina56080414

**Published:** 2020-08-16

**Authors:** Myoungsuk Kim

**Affiliations:** College of Nursing, Kangwon National University, Gangwon-Do 24341, Korea; cellylife@gmail.com; Tel.: +82-33-250-8877

**Keywords:** person-centered care, empathic competence, interpersonal competence, nursing students, perceived stress

## Abstract

*Background and objectives:* Numerous theoretical and clinical advances have been made through research on person-centered care (PCC). Nevertheless, care is still focused on the medical aspects of treating patients’ diseases in Korea, and thus providing individualized PCC to patients tends to be neglected. This study aimed to investigate the relationship between PCC competence, empathic competence, interpersonal competence, and perceived stress to identify the factors that impact PCC competence for developing programs that foster PCC competence in nursing students. *Materials and Methods:* Data were collected from 149 participants, which comprised third- and fourth-year nursing students from two universities in Korea who have experienced clinical training. PCC competence, empathic competence, interpersonal competence, and perceived stress were measured using structured self-reported questionnaires. *Results*: PCC competence was positively correlated with empathic competence (*p* < 0.001) and interpersonal competence (*p* < 0.001), and negatively correlated with perceived stress (*p* < 0.001). Empathic competence, perceived stress, interpersonal competence, and satisfaction with the participants’ nursing major were identified as factors that influenced the PCC competence (adjusted R^2^ = 0.570). *Conclusions:* To enhance PCC competence in nursing students, empathic competence, interpersonal competence, and satisfaction with the participants’ nursing major need to be improved and perceived stress needs to be reduced.

## 1. Introduction

Person-centered care (PCC) refers to individualized care provided by medical and other care professionals through a therapeutic relationship with patients [1]. It is characterized by care providers focusing more on the person as opposed to the illness, respecting and actively responding to patients, and ensuring patients’ rights and autonomy are upheld in treatment-related decision making. Thus far, patient-centered care and PCC have been used interchangeably [2]. Although these two concepts share a number of common aspects, such as empathy, respect, engagement, holistic focus, relationship, communication, and shared decision-making, they differ in that the goal of patient-centered care is to live a functional life, such as pain reduction, whereas that of PCC is for a person to live a “meaningful life” [3]. Today, the focus is being shifted from patient-centered care to PCC where patients are viewed beyond the disease-focused medical perspective as people with preferences and needs [4]. Further, PCC is a concept that expands the perspective of patient-centered care in consideration of patients’ whole lives [3].

Numerous theoretical and clinical advances have been made worldwide through research on person-centered care [5]. Nevertheless, care is still focused on addressing medical problems in Korea, and thus providing individualized care to patients tends to be neglected [6]. Moreover, the nursing curriculum for Korean nursing students does not include content related to developing PCC. Since Korea’s nursing education has been generally focused on care in acute-care hospital environments, measures used to enhance PCC should be explored and incorporated into nursing curricula [7]. In addition, research on PCC among nursing students is limited to qualitative studies or the introduction of educational approaches [8,9], rather than the study of intervention programs that improve PCC conducted by nurses [6]. As nursing students are equipped with knowledge as well as professional values and attitudes through theoretical education and clinical training, nursing curricula should be updated to provide humanistic and holistic care through the cultivation of knowledge and attitudes for PCC.

Therefore, identifying the factors that impact PCC in nursing students is important in order to incorporate PCC education into the nursing curriculum. In order to form a trust relationship based on PCC with patients, nurses are required to have empathy, which enables them to adequately understand the circumstances, perspectives, and psychological needs of patients [10]. Interpersonal competence is another critical attribute required for practicing PCC to build good interpersonal relationships with patients and other healthcare professionals in clinical practice [11]. In addition, nursing students tend to perceive higher levels of stress compared to students in other majors, as they undergo an overwhelming amount of theoretical education and clinical training [12]. Their perceived stress contributes to diminished clinical performance during clinical training [13], and high perceived stress leads to self-centered thinking as a result of losing self-regulation and prevents altruistic social interest behaviors that are helpful to others [14]. Hence, perceived stress may be a barrier to PCC in nursing students, but studies attempting to investigate the factors that influence PCC are lacking.

This study aimed to investigate the level of PCC competence among nursing students and to examine the impact of not only empathic competence and interpersonal competence but also perceived stress, which is a barrier for practicing PCC, to lay the foundation for developing programs that foster PCC competence among nursing students.

## 2. Materials and Methods

### 2.1. Study Design

This study was a descriptive survey that aimed to examine the levels of and correlations between PCC competence, empathic competence, interpersonal competence, and perceived stress, as well as the impact of empathic competence, interpersonal competence, and perceived stress on PCC competence in nursing students.

### 2.2. Participants

Third- and fourth-year nursing students attending one of two nursing colleges in Chuncheon city, Gangwon-Do, Republic of Korea, were recruited. The inclusion criteria comprised third- and fourth-year nursing students who had completed at least one semester of clinical training. Participants voluntarily responded to a recruitment poster and signed a written consent form. The appropriate sample size was computed using G*-Power software (G*-Power 3.1.9.2, Heinrich-Heine-University, Düsseldorf, Germany). By considering the six demographic characteristics and three independent variables to be studied, the maximum number of independent variables for the sample size calculation was set to be nine. For a multiple regression with a significance level of 0.05, moderate effect size of 0.15, power of 0.90, and nine independent variables, the minimum required sample size was 141. Based on a 10% withdrawal rate, 150 questionnaires were distributed and all of them were retrieved. After excluding one questionnaire with incomplete responses, 149 were included in the final analysis.

### 2.3. Instruments

#### 2.3.1. PCC Competence

PCC competence was assessed using an instrument developed by Suhonen and Gustafsson [15] and adapted into Korean for use on nursing students by Park [16]. The instrument consisted of 17 items, with each item rated on a five-point Likert scale, whereby a higher score indicates higher PCC competence. The Cronbach’s α was 0.89 in Park’s [16] study and 0.86 in this study.

#### 2.3.2. Empathic Competence

Empathic competence was assessed using the empathic competence scale developed by Lee and Seomun [17]. The instrument consists of 17 items, with each item rated on a five-point Likert scale, whereby a higher score indicates greater empathic competence. The Cronbach’s α was 0.93 in the study by Lee and Seomun [17] and 0.93 in this study.

#### 2.3.3. Interpersonal Competence

Interpersonal competence was assessed using an instrument developed by Buhrmester and Furman [18], and adapted into Korean and validated for use among college students by Han and Lee [19]. The instrument consists of 31 items, with each item rated on a five-point Likert scale. A higher score indicates greater interpersonal competence. The Cronbach’s α was 0.84 in the study by Han and Lee [19] and 0.85 in this study.

#### 2.3.4. Perceived Stress

Perceived stress was assessed using the Perceived Stress Scale (PSS) developed by Cohen and Kamarck [20], and was adapted into Korean and validated for use on college students by Park and Seo [21]. The instrument consists of 10 items, with each item rated on a five-point Likert scale. Of the 10 items, four positive items were reverse-scored, and a higher total score indicated greater perceived stress. The Cronbach’s α was 0.76 in the study by Park and Seo [21] and 0.78 in this study.

### 2.4. Data Collection and Ethical Considerations

Data were collected from 1 September to 30 September 2019. The recruitment advertisement was posted on the bulletin board of the nursing department. Nursing students who voluntarily responded to the recruitment ad were given a verbal explanation of the purpose of the study and a guarantee of anonymity by a research assistant. To control for exogenous variables that may occur when a researcher conducts a survey, a research assistant distributed the questionnaires to the participants who consented and instructed them to drop the completed questionnaires in a dropbox placed in the nursing department’s office after they completed the questionnaires individually in a place and time of their choice. The participants received a coupon of about $5 after the survey was completed.

### 2.5. Ethical Considerations

Prior to data collection, this study was approved by the institutional review board at Kangwon National University (IRB No.: KWNUIRB-2019-05-003-001). The participants voluntarily signed up to participate in the study and they were given a verbal and written explanation of the purpose of the study before completing the questionnaire. They were informed that the collected data would remain anonymous, would be discarded after the study’s completion, would only be used for research purposes, and that all contents in the questionnaire would be coded to remove any personally identifiable information. In addition, the participants were informed that they could withdraw from the study at any time while filling out the questionnaire.

### 2.6. Statistical Analysis

The collected data were statistically processed using IBM SPSS Statistics 23.0 software (IBM Corp., Armonk, NY, USA). Participants’ demographic characteristics, PCC competence, empathic competence, interpersonal competence, and perceived stress were analyzed with descriptive statistics, such as the frequency with a percentage and mean with a standard deviation. The reliability of the instruments for PCC competence, empathic competence, interpersonal competence, and perceived stress was analyzed to provide Cronbach’s α values [16]. The differences in PCC competence according to study participants’ demographic characteristics were analyzed using independent *t*-tests and one-way analysis of variance. The correlations between PCC competence, empathic competence, interpersonal competence, and perceived stress were analyzed using Pearson correlation coefficients [16]. The impact of empathic competence, interpersonal competence, and perceived stress on PCC competence was analyzed with a multiple regression analysis [16,22].

## 3. Results

### 3.1. PCC Competence According to Demographic Characteristics

To identify the demographic characteristics, age, gender, religion, overall GPA (maximum 4.5) in the preceding semester, and satisfaction with their nursing major were surveyed. Table 1 shows the differences in PCC competence according to these demographic characteristics. The majority of the participants were under the age of 25 (70.5%), third-year nursing students (77.9%), and women (79.2%). Most participants (56.4%) followed no religion, and the most common academic score in the preceding semester was 3.0–3.9 (69.1%). A total of 47.7% of the nursing students claimed to be satisfied with their nursing major. PCC competence significantly differed according to satisfaction with nursing major, where those who were satisfied with their nursing major showed higher PCC competence than those who claimed to be neutral or dissatisfied with their nursing major (F = 9940, *p* < 0.001).

### 3.2. Level of PCC Competence, Empathic Competence, Interpersonal Competence, and Perceived Stress

Table 2 shows the levels of PCC competence, empathic competence, interpersonal competence, and perceived stress in the study participants. The mean PCC competence score was 3.61 ± 0.56 out of 5, and the mean empathic competence score was 3.69 ± 0.64 out of 5. The mean interpersonal competence score was 3.34 ± 0.47 out of 5, and the mean perceived stress score was 1.58 ± 0.63 out of 4.

### 3.3. Correlations between PCC Competence, Empathic Competence, Interpersonal Competence, and Perceived Stress

Table 3 shows the correlations between PCC competence, empathic competence, interpersonal competence, and perceived stress. There was a strong positive correlation between empathic competence and PCC competence (*r* = 0.703, *p* < 0.001) and between interpersonal competence and PCC competence (*r* = 0.400, *p* < 0.001). There was a negative correlation between perceived stress and PCC competence (*r* = −0.534, *p* < 0.001).

### 3.4. Factors Influencing PCC Competence

A multiple regression analysis was performed to identify the factors that affected PCC competence (as seen in Table 4). The nominal variables were dummy-coded. The results showed that the regression model was significant (F = 40.259, *p* < 0.001). Homoscedasticity, normality, and multicollinearity were also analyzed. The range of tolerance was 0.75–0.99 and that of the variation inflation factor (VIF) was 1.01–1.45, confirming there was no risk of multicollinearity between the independent variables. Further, the obtained Durbin–Watson statistic was 1.87, confirming the absence of autocorrelation between the error terms. The adjusted R^2^ was 0.570, meaning that the measured variables explained 57.0% of the variance in PCC competence. Empathic competence had the greatest impact on PCC competence in participants (*β* = 0.512, *p* < 0.001), followed by perceived stress (*β* = −0.213, *p* < 0.001), interpersonal competence (*β* = 0.135, *p* = 0.023), satisfaction with nursing major dummy 1 (*β* = −0.146, *p* = 0.011), and satisfaction with nursing major 2 (*β* = −0.118, *p* = 0.047).

## 4. Discussion

This study attempted to investigate the impact of empathic competence, interpersonal competence, and perceived stress on the PCC competence of nursing students in order to present foundational data for developing an effective program to foster PCC competence in nursing students.

In terms of the differences in PCC competence according to participants’ demographic characteristics, the PCC competence significantly differed according to satisfaction with the nursing students’ study major. In other words, nursing students who claimed to be satisfied with their nursing major demonstrated higher PCC competence than those who claimed to be neutral or dissatisfied with their nursing major. This is consistent with the results of a previous study on the factors that affect PCC competence in nursing students [16], where PCC competence differed according to satisfaction with the nursing students’ study major. As suggested by the result, satisfaction with the nursing students’ study major affects empathy in nursing students [22] and PCC competence seemed to differ according to satisfaction with the nursing students’ study major due to its impact on empathy [3], which is an important concept for PCC.

Regarding the mean scores for PCC competence, empathic competence, interpersonal competence, and perceived stress, the overall mean PCC competence score was 3.61 ± 0.56 out of 5. This was lower than that measured for fourth-year nursing students using the same instrument (3.83 ± 0.48) [16]. In this study, the mean PCC competence score for third-year nursing students was 3.56 ± 0.55, and that for fourth-year nursing students was 3.78 ± 0.57. Although statistically insignificant, fourth-year nursing students showed higher PCC competence scores. Factors that impact PCC competence by study year would be helpful for developing customized PCC-strengthening programs.

The mean empathic competence score was 3.69 ± 0.64 out of 5, which was similar to that found in third- and fourth-year nursing students with clinical training experience (3.60 ± 0.33) [23]. Empathy is important for nursing students to provide person-centered care during clinical practice, to provide high-quality nursing care to patients, and to establish a therapeutic interpersonal relationship with patients [3,10,24]. The mean interpersonal competence score was 3.34 ± 0.47 out of 5, which was similar to that found in second- and third-year nursing students (3.38 ± 0.46) [25]. The mean perceived stress score was 1.58 ± 0.63, which was lower than that found in third-year nursing students (1.86 ± 0.56) [26]. Because nursing students show higher perceived stress compared to college students of other majors [12], further studies are needed to determine whether this is attributable to the school year, gender, or other factors.

Regarding the correlations between PCC competence, empathic competence, interpersonal competence, and perceived stress, PCC competence was higher with greater empathic competence, higher interpersonal competence, and lower perceived stress. Empathic competence is an important aspect of PCC [10], and this result is consistent with a previous finding of a study conducted in nursing students, suggesting that empathy is positively correlated with PCC competence [16]. Although we cannot compare the present result on interpersonal competence and PCC competence due to a lack of relevant studies, interpersonal competence is essential for PCC [11] because individualized and holistic care in PCC is based on therapeutic interpersonal relationships [2]. In this study, PCC competence increased with decreasing perceived stress. A previous study reported that when the perceived stress is high, people engage in self-centered thinking, as opposed to focusing on the patient [14]. The most common stressor for nursing students during clinical training was found to be the lack of knowledge and professional abilities to care for patients [27]. Perceived stress caused by this factor during clinical training serves as a barrier for PCC; therefore, lowering perceived stress is crucial when developing PCC-strengthening programs for nursing students.

The factors that influenced PCC competence in nursing students were empathic competence, interpersonal competence, perceived stress, and satisfaction with their nursing major. This is consistent with the previous finding of a study conducted on nursing students, suggesting that the acceptance of perspective, a component of empathy, was a significant factor of PCC [16]. As empathy is an essential component of PCC [3], increasing nursing students’ empathy is crucial. However, nursing students were found to demonstrate less empathy than college students in other health professions [13]. In order to improve PCC competence in nursing students, it is necessary to promote empathy through educational programs [22]. As shown here, empathy, which allows a person to accept and recognize the perspectives of others, is essential to PCC since it aims to provide respectful and individualized care and allow for patient involvement in decision-making.

Next, as demonstrated by findings from this research project, perceived stress also influenced PCC competence. The present results cannot be compared with previous findings due to the lack of studies showing that perceived stress is a factor affecting PCC competence in nursing students. However, nursing students must undergo their theoretical education in addition to clinical training, and they were reported to have higher levels of stress during clinical training than during regular lectures [13]. As shown here, strategies to reduce nursing students’ perceived stress during clinical training are important when developing PCC strengthening programs.

Interpersonal competence also influenced PCC competence, which is important given that therapeutic relationships with patients are an important aspect of PCC [2,3]. Interpersonal competence refers to the ability to remain peaceful with people encountered through work by effectively resolving issues and handling conflicts [28]. PCC-strengthening programs should be included in education and training to improve interpersonal competence in order to help nursing students maintain a good relationship with patients and fellow professionals in clinical practice.

Finally, satisfaction with the nursing major was found to influence participants’ PCC competence. Nursing students’ satisfaction with their nursing major refers to their major being in line with their expectations for a future career, and nursing students tend to form positive values when satisfaction with their study major is high [29]. Furthermore, nursing students that feel high satisfaction with their nursing major demonstrated a higher level of empathic competence than those who were dissatisfied with their nursing major [24]. To enhance PCC competence, interventions that can boost nursing students’ satisfaction with their nursing major are needed.

This study had several limitations. First, the participants were nursing students at two nursing colleges in Chuncheon city, Republic of Korea, which makes it difficult to generalize our findings to all nursing students. Second, the instrument of empathic competence used in this study was developed in Korean; therefore, it might have limited replicability at an international level.

## 5. Conclusions

This study examined the relationships between PCC competence, empathic competence, interpersonal competence, and perceived stress, and identified the impact of empathic competence, interpersonal competence, and perceived stress on PCC competence. PCC competence increased with increasing empathic competence, increasing interpersonal competence, and decreasing perceived stress. Furthermore, empathic competence, perceived stress, interpersonal competence, and satisfaction with their study major were identified as the factors that influenced PCC competence in nursing students who participated in this study.

These results suggest that empathic competence, perceived stress, interpersonal competence, and satisfaction with the nursing major must be considered when developing programs to foster PCC competence between nursing students. In addition, the study can be expected to bring about changes in clinical nursing by improving PCC competence for nursing students who will become nurses in Korea. Based on these results, it is suggested that subsequent studies develop and assess the effects of an intervention program for enhancing PCC competence in nursing students by taking empathic competence, perceived stress, interpersonal competence, and satisfaction with the nursing major into consideration. Furthermore, studies should also analyze the different factors that are in play regarding the nursing students’ study year in order to develop tailored PCC interventions for nursing students at varying levels of advancement in their nursing major. Finally, this study should be replicated to more accurately identify the various predictors of PCC competence.

## Figures and Tables

**Table 1:** Differences in the person-centered care (PCC) competence by demographic characteristics (*n* = 149).

Characteristics	Categories	*n* (%)	Mean ± Standard Deviation	*t* or F (*p*)	Scheffé
Age (years)	<25	105 (70.5)	58.70 ± 10.24	−1.552 (0.122)	
	≥25	44 (29.5)	61.34 ± 7.18		
Grade	Third-year	116 (77.9)	60.45 ± 9.50	−1.784 (0.076)	
	Fourth-year	33 (22.1)	64.27 ± 9.70		
Gender	Female	118 (79.2)	60.05 ± 9.73	−1.431 (0.155)	
	Male	31 (20.8)	57.32 ± 8.32		
Religion	Yes	65 (43.6)	57.86 ± 9.47	−1.846 (0.066)	
	No	84 (56.4)	60.73 ± 9.37		
Overall GPA in the preceding semester	<3.0	15 (10.1)	63.20 ± 8.72	1.483 (0.230)	
	3.0–3.9	103 (69.1)	59.34 ± 9.72		
	≥4.0	31 (20.8)	58.13 ± 8.90		
Satisfaction with the nursing major	Satisfied	71 (47.7%)	63.23 ± 8.64	9.940 (<0.001)	Satisfied > Neutral, Dissatisfied
	Neutral	66 (44.3%)	58.86 ± 8.39		
	Dissatisfied	12 (8.1%)	52.75 ± 7.89		

Data are expressed as mean ± standard deviation or as *n* (%). Tested by independent *t*-tests or one-way analysis of variance.

**Table 2:** Mean scores of PCC competence, empathic competence, interpersonal competence, and perceived stress (*n* = 149).

Variables	Mean ± Standard Deviation	Range	Actual Range	
			Min	Max
PCC competence	3.61 ± 0.56	1–5	2.06	5.00
Empathic competence	3.69 ± 0.64	1–5	1.70	4.88
Interpersonal competence	3.34 ± 0.47	1–5	1.61	4.54
Perceived stress	1.58 ± 0.63	0–4	0.50	3.30

**Table 3:** Correlation between PCC competence, empathic competence, interpersonal competence, and perceived stress (*n* = 149).

Variables	PCC Competence	Empathic Competence	Interpersonal Competence
	*r* (*p*)	*r* (*p*)	*r* (*p*)
Empathic competence	0.703 (<0.001)		
Interpersonal competence	0.400 (<0.001)	0.362 (<0.001)	
Perceived stress	−0.534 (<0.001)	−0.491 (<0.001)	−0.245 (0.003)

Tested by Pearson correlation coefficients.

**Table 4:** Factors influencing PCC competence in nursing students (*n* = 149).

Variables	B	Standard Error	*β*	95% Confidence Interval	*t*	*p*
(Constant)	30.146	5.470		19.849 to 41.341	5.512	<0.001
Empathic competence	0.449	0.057	0.512	0.336 to 0.562	7.874	<0.001
Perceived stress	−0.318	0.094	−0.213	−0.505 to −0.132	−3.372	0.001
Interpersonal competence	0.088	0.038	0.135	0.012 to 0.164	2.300	0.023
Satisfaction with nursing major dummy 1 *	−2.824	1.090	−0.146	−4.994 to −0.653	−2.572	0.011
Satisfaction with nursing major dummy 2 *	−4.175	2.080	−0.118	−8.300 to 0.050	−2.001	0.047
R^2^ = 0.585, Adjusted R^2^ = 0.570, F = 40.259 (*p* < 0.001)						

* Dummy variable: satisfaction with nursing major (0—satisfaction); tested using linear multiple regression analysis.
